# A Case of Poisoning by Muriate of Cocaine

**Published:** 1885-10

**Authors:** G. W. Kennicott

**Affiliations:** 90 Thirty-third street


TO THE EDITORS OF THE *Chicago Medical Journal and Examiner:*

*Gentlemen:*

The following case of poisoning by *muriate of cocaine* may prove interesting to your readers:

Mrs. S., age twenty-five, of good constitution, has been using a two per cent. preparation of cocaine, prescribed for hay-fever by a well-known physician of this place, with good success. September 2d, having exhausted the vial, her husband,

*without a prescription*, procured from a druggist two five-grain vials of the muriate of cocaine, full strength, of which Mrs. S., about 5 o'clock P. M., applied two-thirds of the contents of one bottle to both nostrils with a small glass insufflator. In fifteen or twenty minutes she became dizzy, her vision became dark, a sinking sensation occurred, with great weakness.

At 5.30 P. M. I was called. I found the patient in semi-comatose condition, from which she was easily aroused, but answered questions with difficulty. When so aroused, her mind was clear. Her temperature was above normal. Her skin was hot and dry. The radial pulse was very rapid, and so weak as to be scarcely discernible. Her pupils were widely dilated. Deglutition and articulation were difficult. There was some dyspnœa. She complained of dryness of her fauces and a bitter taste in her mouth. She complained also of cold shivers, and her teeth chattered, although her temperature was still above normal. Later she became drowsy; her eyes were closed and the muscles of her face affected. There was great weakness; she could not support her head. There was dyspnœa, but it was not severe. There was some nausea, but she did not vomit. Her extremities were cool and her mind clear when aroused.

She recovered in about three hours under stimulants (brandy and ammonia) and digitalis. Heat and friction were applied to her extremities, and heat over the epigastrium.

On September 3d, at 9 A. M., I called to see patient. She was feeling well and noticed no ill-effects from the drug.

90 Thirty-third street. Yours respectfully,

G. W. KENNICOTT, M.D.

September 4th.

